# A commentary on frontostriatal salience network expansion in individuals in depression

**DOI:** 10.1093/psyrad/kkaf005

**Published:** 2025-03-17

**Authors:** Sarah Katharina Buehler, Ruibin Zhang, Jonathan Roiser

**Affiliations:** Institute of Cognitive Neuroscience, University College London, London, UK; Laboratory of Cognitive Control and Brain Healthy, Department of Psychology, School of Public Health, Southern Medical University, Guangzhou, PR China; Department of Psychiatry, Zhujiang Hospital, Southern Medical University, Guangzhou, PR China; Institute of Cognitive Neuroscience, University College London, London, UK

A key goal of neuroimaging research in depression is the discovery of neural markers, such as patterns of brain structure or function, that can identify individuals with or at risk of developing symptoms (Tozzi *et al*., 2024). While most functional magnetic resonance imaging (fMRI) studies are cross-sectional and use group-average parcellations to define brain networks, major depressive disorder (MDD) is heterogeneous, with potentially clinically informative individual differences in brain organization unaccounted for by standard analytic approaches.

In their study, Lynch *et al*. (2024) used fMRI to collect an impressive dataset of densely sampled (up to 62 times over 1.5 years) resting-state scans in individuals diagnosed with MDD. They applied 'precision functional mapping', using the Infomap community detection algorithm, which identifies brain network topology within each individual, as opposed to typical group-average parcellations, to delineate 20 functional networks. They then investigated differences in network size relative to a non-depressed group, by computing the percentage of cortical surface area occupied by each network.

The key finding is a substantially enlarged 'salience network' (incorporating the lateral prefrontal and dorsal anterior cingulate cortex, anterior insula, and striatum) in MDD relative to a non-depressed control group. This was initially identified in a very small discovery sample (*n* = 6 depressed, *n* = 37 controls), detecting an extremely large effect size (standardized mean difference; Cohen's *d* = 1.99). Importantly, this finding was replicated in three independent datasets (*n* = 42, 45, 48) with substantially smaller, but still large, effect sizes (Cohen's *d* = 0.77–0.84), as well as in large previously published datasets, where precision mapping was applied to group-averaged instead of individual-level data (although statistical group differences were unfortunately not reported).

There are important considerations for future attempts to replicate these promising findings. As with most advanced neuroimaging analysis pipelines, it remains unclear how sensitive the results are to methodological choices applied during the precision functional mapping procedure, such as statistical thresholding and preprocessing of input connectivity matrices. For example, no significant group difference in salience network representation was found in the striatum, but there was extreme variability in the percentage of the striatum occupied by the salience network between individuals (0–80%, Fig. 1d in Lynch *et al*. (2024)), even within the healthy control sample. This raises concerns that the mapping of the network in this region may have been imprecise and introduced substantial noise.

**Figure 1:**
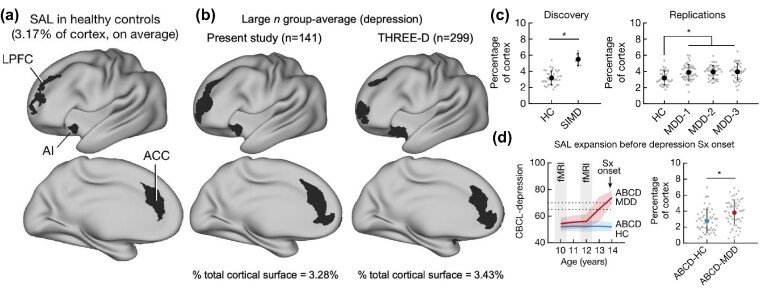
Frontostriatal salience network expansion in depression. (a) The frontostriatal salience network has representations in the lateral prefrontal cortex (LPFC), dorsal anterior cingulate cortex (ACC), anterior insula (AI), and striatum. This network occupied on average 3.17% of the total cortical surface in healthy controls in the discovery sample (*n* = 37), while (b) it was expanded to an average of 3.28% of the cortex in the discovery depression sample and three initial replication datasets combined (*n* = 6, 42, 45, and 48, total *n* = 141), and 3.43% in a previously published dataset (*n* = 299). (c) The differences were very large when comparing the healthy control group (HC) to the initial discovery sample (SIMD) [standardized mean difference (Cohen's *d* = 1.99)], and large when comparing to the three replication datasets (MDD-1, MDD-2, MDD-3) (Cohen's *d* = 0.77–0.84). (d) In a cohort study the salience network was already expanded, 36% on average, in 10–11-year-olds who later developed clinically significant symptoms of depression (ABCD-MDD, *n* = 57), relative to their non-depressed peers (ABCD-HC, *n* = 57) (Cohen's *d* = 0.62). Figure assembled from figures in Lynch *et al*. (2024).

Another consideration concerns the quantity and quality of fMRI data required to detect differences in network size. The authors attribute their discovery to the uniquely dense sampling and mapping of functional networks within individuals. Supplementary analyses of their discovery sample suggest that with less than 2 h of fMRI data an ∼20% lower effect size was observed. However, network expansion was detectable in several replication datasets, which each acquired only around 1 h of fMRI data, or even used group-level data. This suggests that neither extended fMRI sequences nor individual-level mapping of functional networks is absolutely necessary to observe salience network expansion in MDD.

Lynch and colleagues also conducted a classification analysis, finding that depressed individuals (*n* = 144) were distinguishable from healthy controls (*n* = 37) with almost 80% accuracy based on the size of all 20 networks, with salience network size emerging as the most discriminating feature. While this classification accuracy is impressive, it is common for machine learning models trained on small and highly unbalanced samples to subsequently fail to replicate in completely independent datasets (Chekroud *et al*., 2024). Given that this analysis included nearly four times as many depressed patients than controls, the observed classification accuracy may well be inflated, and consequently the robustness of this result requires confirmation in future studies.

The authors also investigated several features of salience network size, demonstrating that it remains stable over time, is independent of symptom fluctuations, and is unaffected by repetitive transcranial magnetic stimulation (rTMS) treatment (although no clinical outcomes were reported to contextualize this result). Overall, these findings support the authors’ interpretation of salience network size as a trait-like marker.

This conclusion is reinforced by the paper's most striking result. The authors included developmental cohorts from the Adolescent Brain Cognitive Development (ABCD) study, whose depression scores were assessed every year between the age of 10–14 years, with fMRI scans at age 10 and again at 12 years. Remarkably, the salience network was already expanded, by 36% on average, in children who later (aged 13 or 14 years) developed clinically significant depression symptoms (*n* = 57), relative to their non-depressed peers (*n* = 57). While this suggests salience network expansion may represent a promising risk-marker, it would be substantially more informative if the authors had matched their selected sub-sample to a control group based on the rich socio-demographic and environmental factors available in ABCD (e.g. early-life stress), or at least included these variables as covariates, as the root cause of salience network expansion remains unclear.

Several questions remain unresolved with respect to the clinical utility of salience network expansion in MDD. First, it was noticeable that average salience network size, in terms of percentage of cortical surface area occupied, differed markedly between the healthy control groups in the discovery (3.17%) and large replication datasets (1.98%, 1.27%). This marked variation casts doubt on whether there is a clinically meaningful threshold for salience network size, or if expansion in MDD might be driven by inclusion criteria for the recruited control groups, which could potentially inflate group differences. Second, high degrees of variability in salience network size were evident within MDD groups, yet these individual differences were unrelated to depression severity. Third, as alluded to above, it is unclear whether enlarged salience networks are relevant to the aetiology of depression. One possibility is that the expanded salience network is a consequence of some risk factor for depression, but not causally involved in the generation of symptoms. This would also explain why salience network size was unrelated to variability in symptoms, either within or between individuals. While the salience network is implicated in several cognitive processes relevant to depression, such as processing affectively salient stimuli and emotional regulation, it is unclear how these functions relate to the network's size. Future work using task-based fMRI could test whether experimental manipulations can perturb salience network size or identify cognitive processes impacted by an alteration in its size. Finally, an important feature of reliable clinical markers is disorder specificity. The authors reported preliminary analyses in which differences in salience network size were not evident in autism spectrum disorder (ASD) or obsessive-compulsive disorder (OCD) but were detectable in mild bipolar disorder. However, larger samples are needed to robustly assess specificity.

In summary, Lynch and colleagues discovered that salience network expansion may be a promising trait-like marker for depression, present even prior to the onset of symptoms. Importantly this marker was unrelated to depression severity either between or within subjects, unresponsive to (rTMS) treatment, and not evident in ASD or OCD. These results pave the way for advancing the field of precision psychiatry. We hope that future work will attempt independent replications of salience network expansion, as well as probing its disorder specificity and underlying mechanisms. The extent to which salience network expansion is related to environmental or genetic risk factors for depression also requires further investigation.

## References

[bib1] Chekroud AM, Hawrilenko M, Loho H et al. (2024) Illusory generalizability of clinical prediction models. Science. 383:164–7. 38207039 10.1126/science.adg8538

[bib2] Lynch CJ, Elbau IG, Ng T et al. (2024) Frontostriatal salience network expansion in individuals in depression. Nature. 633:624–33. 39232159 10.1038/s41586-024-07805-2 PMC11410656

[bib3] Tozzi L, Zhang X, Pines A et al. (2024) Personalized brain circuit scores identify clinically distinct biotypes in depression and anxiety. Nat Med. 30:2076–87. 38886626 10.1038/s41591-024-03057-9 PMC11271415

